# Targeted Chiral Metabolomics of D-Amino Acids: Their Emerging Role as Potential Biomarkers in Neurological Diseases with a Focus on Their Liquid Chromatography–Mass Spectrometry Analysis upon Chiral Derivatization

**DOI:** 10.3390/ijms252212410

**Published:** 2024-11-19

**Authors:** Cinzia Lella, Liam Nestor, Dimitri De Bundel, Yvan Vander Heyden, Ann Van Eeckhaut

**Affiliations:** 1 Research Group Experimental Pharmacology (EFAR), Center for Neurosciences (C4N), Vrije Universiteit Brussel (VUB), Laarbeeklaan 103, 1090 Brussels, Belgium; cinzia.lella@vub.be (C.L.); liam.nestor@vub.be (L.N.); dimitri.de.bundel@vub.be (D.D.B.); 2 Research Group Analytical Chemistry, Applied Chemometrics and Molecular Modelling (FABI), Vrije Universiteit Brussel (VUB), Laarbeeklaan 103, 1090 Brussels, Belgium; yvan.vander.heyden@vub.be

**Keywords:** D-amino acids, biomarkers, neurological diseases, chirality, enantioselective analysis, LC-MS/MS, chemical derivatization

## Abstract

In neuroscience research, chiral metabolomics is an emerging field, in which D-amino acids play an important role as potential biomarkers for neurological diseases. The targeted chiral analysis of the brain metabolome, employing liquid chromatography (LC) coupled to mass spectrometry (MS), is a pivotal approach for the identification of biomarkers for neurological diseases. This review provides an overview of D-amino acids in neurological diseases and of the state-of-the-art strategies for the enantioselective analysis of chiral amino acids (AAs) in biological samples to investigate their putative role as biomarkers for neurological diseases. Fluctuations in D-amino acids (D-AAs) levels can be related to the pathology of neurological diseases, for example, through their role in the modulation of N-methyl-D-aspartate receptors and neurotransmission. Because of the trace presence of these biomolecules in mammals and the complex nature of biological matrices, highly sensitive and selective analytical methods are essential. Derivatization strategies with chiral reagents are highlighted as critical tools for enhancing detection capabilities. The latest advances in chiral derivatization reactions, coupled to LC-MS/MS analysis, have improved the enantioselective quantification of these AAs and allow the separation of several chiral metabolites in a single analytical run. The enhanced performances of these methods can provide an accurate correlation between specific D-AA profiles and disease states, allowing for a better understanding of neurological diseases and drug effects on the brain.

## 1. Introduction

In recent years, metabolomics has emerged as an extremely useful tool in the field of medical sciences. Defined as the analysis of all free low-molecular-weight metabolites (≤1500 Da) involved in biochemical reactions within a biological system [1,2,3], metabolomics provides valuable insights into the phenotype of a cell or tissue and its changes due to environmental or genetic causes [4,5]. Furthermore, the metabolome is considered as the downstream product of the proteome, encompassing the comprehensive set of by-products of gene transcription, posttranslational modifications, and enzymatic reactions. Minor alterations in enzyme concentrations can significantly influence the concentration of free metabolic intermediates, thus impacting the cell’s phenotype. Consequently, metabolomics findings offer an indicative snapshot of the status of the biological system of interest [6,7]. Since the other “omics” sciences within life sciences, namely genomics, transcriptomics, and proteomics, cannot provide a complete overview of the in-vivo enzymatic activity, metabolomics efficiently contributes through the detection and quantification of free metabolites. From the metabolic profile, a better understanding of biochemical reaction fluxes and networks can be achieved [3].

Metabolites serve as an extremely sensitive measure of cell conditions, since the metabolome is more responsive to perturbations than the transcriptome or proteome [8]. Metabolic intermediates and end products are also more easily accessible in vivo, enabling real-time assessment of the health of the tissue of interest [5]. Thanks to the actual advances in analytical methods, their changes can be measured within minutes, while alterations in protein or mRNA expression typically take longer to be detected [9]. Another advantage related to metabolomic studies is the higher conservation of metabolites across species, i.e., from bacteria to complex organisms, allowing for easier correlations between metabolomics studies in model organisms and human data [5].

In this review, the focus is on chirality, a recurrent feature in biological processes. The metabolome is composed of a complex set of biomolecules with a variety of chemical features. Achiral and chiral compounds are both involved in catabolism and anabolism. In view of this, enantioselective metabolomics is gaining more and more relevance in the last few decades. For a long time, the metabolome was believed to be mainly homochiral, relying on the preponderance of one enantiomer [10]. Notably, D-amino acids (D-AAs) were thought to be unnatural; they were rarely found in living organisms as free metabolites or as elements of peptides or proteins. On the other hand, L-amino acids (L-AAs) are recognized as the main form of free AAs and as the quintessential building blocks of peptides and proteins. Therefore, metabolomics had relied only on achiral methods. However, a change of course has been made with the development of sensitive analytical methods, for instance, through hyphenated liquid chromatography–mass spectrometry (LC-MS) set-ups. The first stone laid was the discovery of the interaction of endogenous D-serine (D-Ser), D-aspartate (D-Asp), and D-alanine (D-Ala) with the N-methyl-D-aspartate receptor (NMDAR) complex in the rat brain [11,12]. This paved the way for a new evaluation of the role and the relevance of D-AAs in mammals, together with the further development of chiral analytical methods [12,13,14]. In this sense, a valuable strategy for chiral resolution is the pre-column derivatization of the sample with a chiral derivatization reagent (CDR), allowing the separation of several DL-AAs in a single analytical run [15].

This review provides an overview of D-amino acids in neurological diseases and of the state-of-art strategies based on chiral derivatization for the enantioselective analysis of chiral amino acids in biological samples, to investigate their putative role as biomarkers for neurological diseases.

## 2. Metabolomics Techniques

Two different strategies of analysis are currently employed in metabolomics, namely targeted and untargeted. The untargeted approach is based on the screening of biological samples, leading to the unbiased identification and relative quantification of the metabolome in samples, considering the totality of spectral features. This strategy can be performed for fingerprinting or profiling purposes. Metabolite fingerprinting provides a rapid and high-throughput global sample classification based on the obtained profiles, while metabolite profiling is carried out through the identification and quantification of metabolites, attributed to similar chemistries or metabolic pathways. These two approaches are complementary and can provide a comprehensive characterization and comparison of metabolite patterns, for classification or discrimination aims, across samples that differ in biological origin or status [2,16]. They are widely used, for example, in exploring new biomarkers and identifying global metabolic alterations due to disease or in response to disease treatments. On the other hand, a targeted approach is particularly used for studying specific metabolites of interest, for example, in pre-defined metabolic pathways or for monitoring known biomarkers for certain pathophysiological conditions. It focuses on the absolute quantification of a limited number of predefined metabolites through comparison with standard solutions [6].

Most of the analytical methods in this field rely on nuclear magnetic resonance (NMR) spectroscopy or MS detection. NMR is mainly employed for the untargeted analysis of complex mixtures with little or no sample preparation. Nevertheless, it is characterized by relatively low sensitivity. A well-suited alternative is high-resolution MS, obtained with Fourier transform–ion cyclotron resonance, Orbitrap or time-of-flight mass analyzers. On the other hand, targeted metabolomics is usually performed by tandem MS with a triple quadrupole set-up. However, both approaches generally involve a preceding chromatographic or capillary electrophoretic separation, providing higher resolution and sensitivity [16,17,18,19,20].

Furthermore, “omics” techniques and (statistical) data handling can be combined for more effective and fruitful outcomes. Indeed, bioinformatics and chemometrics are the backbone of metabolomic studies. In other words, since metabolome analysis requires the simultaneous characterization of hundreds of metabolites, rapid consultation of databases and comparison of data are necessary. Hence, several databases have been developed, such as HMDB (human metabolome database) [21], BiGG (database of biochemical, genetic, and genomic metabolic network reconstructions) [22], METLIN (metabolite database) [23], and NIST (spectral database) [24]. In this way, biochemical pathways, compound names and structures, and concentrations in biofluids or tissue locations in the organism of interest are easily consultable, making the metabolite–disease association much easier. Besides, these informatic tools allow for comparison of spectral features of tissues in normal and diseased conditions, simplifying the identification of statistically significant differences between them [5,25,26].

## 3. Brain Metabolomics

Neurons and glial cells (i.e., astrocytes, microglia, and oligodendrocytes) constitute the cellular building blocks of the central nervous system (CNS) and operate at multiple levels. The prime roles of neurons are information processing and transfer, as well as fulfilling motor, sensory, regulatory, and cognitive functions. They are the primary excitable cells of the nervous system. Glial cells regulate metabolic support, modulate synaptic transmission, and serve immune functions [27,28,29]. Neurons and glial cells can both release neuroactive metabolites that can act as neurotransmitters and gliotransmitters, respectively. Examples are glutamate and γ-aminobutyric acid as the most important excitatory and inhibitory transmitters, respectively. Together, neurons and glia are responsible for the characteristic metabolomic profiles in (patho-)physiological conditions of the brain [17,30].

Since the onset of a neurological disease affects the physiological status of a cell, the analysis of metabolites sheds light on the metabolic pathways underlying that pathological condition, leading to the discovery of novel drug targets, but also to the development of new and more efficient treatment strategies and the identification of new (early-stage) biomarkers [1,18].

Nevertheless, brain metabolomics poses unique challenges for the discovery of biomarkers; the complex and vulnerable structure of the brain constitutes an important limitation for in-depth studies of the central nervous system (CNS) and its diseases. In particular, the brain exhibits a broad heterogeneity reflected in phenotypes and interactive connections between neurons and glia that vary widely within the same organ. The physical inaccessibility of this tissue arises from the presence of the skull and the blood–brain barrier (BBB), which protect and surround the brain and make in-depth brain studies challenging because of potential significant and irreversible disruption of their regular functions [31,32]. In this context, for instance, human brain biopsies and manipulations typically involve invasive ante mortem surgical procedures or post mortem tissue sampling, i.e., when the disease has already reached an end stage, thus precluding the investigation of early aetiologies or the accessibility of tissue at the specific site of pathology [31].

Metabolites can be easily detected and quantified in various peripheral tissues, such as blood or plasma, urine, and saliva. However, the BBB limits the passage of several metabolites from the periphery, making it challenging to correlate those found in these biofluids with those actually present in brain extracellular fluid (ECF), cerebrospinal fluid (CSF), or in the rest of the brain [31,33,34]. To overcome this obstacle, specific in vivo sampling techniques have been developed, namely microdialysis, cerebral open-flow microperfusion (cOFM), and solid-phase microextraction (SPME), which are used in preclinical animal studies giving access to ECF and CSF [17,35,36].

## 4. Biomarkers in Neurological Diseases

Neurological diseases are recognized as the primary cause of disability worldwide and the second leading cause of death, following cardiovascular diseases [37]. Their impact poses significant challenges for biomedical research. Between 1990 and 2019, there has been a marked increase in both the prevalence and incidence of neurological disorders. This rise can be attributed to the global increase in life expectancy, which has led to an older population. Additionally, exposure to factors such as environmental pollution and unhealthy lifestyles has further contributed to this trend. In 2019 alone, there were approximately 805 million cases of neurological disorders, with an age-standardized incidence rate of 10,260 per 100,000 people. Notably, significant increases in age-standardized incidence rates were observed in Parkinson’s disease (PD), idiopathic epilepsy, bipolar disorder, Alzheimer’s disease (AD) and other dementias, and headache disorders (migraine) [38,39]. Clearly, there is an urgent global need for new therapies and diagnostic tools as well as higher concern in relation to controlling modifiable risk factors, i.e., smoking, unbalanced diet, and lack of exercise. Furthermore, it is worth noting that prognosis for these patients is still poor. In contrast with the latest advancements in various surgical and pharmacological interventions, most patients receive a diagnosis when nerve damage is already extensive [40].

Recent progresses in metabolomic studies including biomarker discovery are charting a new course for neurological disorders in terms of diagnostics and therapeutics. Establishing a correlation between fluctuations in metabolite profile and disease status, as well as the identification of new potential biomarkers, are possibilities [41]. Even though the underlying mechanisms of these diseases are not clear yet, metabolomics can help monitoring symptoms by focusing on alterations in the brain metabolome, based on animal models in preclinical studies [34].

### 4.1. Biomarker Discovery

The discovery of metabolites as new diagnostic and/or differential biomarkers may enable the identification of disease even at its initial stages, before the onset of symptoms, making possible a personalized treatment pathway and targeted and proficient follow-up after the diagnosis. Moreover, for many diseases, an early start to treatment is beneficial [18].

Many diseases such as AD and PD, as well as epilepsy and neurological disorders in general, are still characterized by a lack of effective therapies, due to insufficient knowledge of the precise underlying mechanisms of the disease and consequently, late detection and diagnosis.

The term ‘biomarker’ encompasses a multitude of concepts referring to different types of objective measurements that can be evaluated as indicators of biological processes in pathophysiological conditions or pharmacological responses to a specific therapy. As a result, they can give insights into the progression of the disease or the effect of therapies, facilitating the classification of a new sample in a more time- and cost-efficient manner [40]. The advent and rise of genomics, transcriptomics, proteomics, and metabolomics have greatly accelerated and simplified this process.

This review focuses on chiral metabolomics as a thriving tool for biomarker discovery in the field of neuroscience. However, metabolomics is not only about identifying and quantifying metabolites but also about attributing a biological meaning to the analytical results, i.e., establishing a link between metabolomic data and metabolism, finding the common thread to link a set of metabolites to the specific biochemical pathway and the physiological outcomes [25].

### 4.2. Free D-Amino Acids in Mammals

Until the second half of the 20th century, it was a common belief that D-enantiomers were exclusively present in bacteria, plants, and invertebrates. This has changed with the development of sensitive hyphenated chiral analytical methods, allowing the detection of D-AAs in mammals. Hence, the first evidence of the presence of free D-AAs in major organisms was obtained in 1986 when free D-Asp was detected in human and rat tissues [42]. This discovery opened the path to the detection of several other D-AAs in mammalian tissues. The presence of these metabolites is influenced by diet, physiological state, age, and medical therapies such as antibiotics administration [43]. D-AAs can be of exogenous origin, ingested through food intake or produced by gut bacteria. For D-Asp, D-Ser, and D-cysteine (D-Cys), an endogenous origin in rodents has also been demonstrated through the presence of specific enzymes that control their production and release, such as serine racemase [44].

#### 4.2.1. Exogenous D-Amino Acids

The dietary origin of D-AAs is a consequence of racemization processes of L-AAs, which can be triggered by extreme pH treatments, heating, or fermentation. The latter derives from microbial activity. Indeed, D-AAs are important components of peptidoglycan layers in bacterial cell walls and can be produced through bacterial amino acid racemases [43,44]. In mammalian intestines, bacteria and polymicrobial clusters are widespread and the gut microbiome represents a major source of D-AAs in animals [43].

D-alanine (D-Ala) was demonstrated in the rat pituitary gland and has been implicated in circadian regulation [45]. In rodents, D-Ala has also been detected in the pineal gland, hippocampus, hypothalamus, cerebellum, midbrain, and pons, while in humans, it was found in white and gray matter, plasma, serum, and CSF [46]. Interestingly, in humans with a normal sleep–wake cycle, increases in serum and urine D-Ala were observed in the late evening, whereas in humans with an opposite sleep–wake profile, a different circadian pattern was detected [47]. D-Ala is derived from diet and microbiota [48], while no endogenous synthesis of D-Ala was observed in germ-free mice [49]. Similarly, D-leucine (D-Leu) was first discovered in the pineal gland and hippocampus, and a role in regulating neuronal excitability and sleep–wake cycles has been proposed [45,46,50]. In particular, its concentration exhibits pronounced day–night fluctuations in plasma and tissues of rats, which are more closely associated with the sleep–wake pattern rather than the light–dark cycle, thus reflecting the activity rhythms of the animals [46,48,51]. A similar trend has been registered for humans [52]. These circadian fluctuations might reflect changes in uptake or breakdown by D-amino-oxidase, given that no endogenous synthesis through D-Ala racemase has been demonstrated [53].

It is generally accepted that D-glutamate (D-Glu) is not produced by higher mammals, but it is present in foods such as soybeans and it is a bacterial cell wall component. Nevertheless, it has been found in high concentrations in rodent kidney and liver, and in smaller amounts in the brain, although these concentrations can vary depending on the specific strain of mice [47,54,55]. D-glutamine (D-Gln) has been found to be the most prominent D-AA in the mouse cortex, while its concentration in the hippocampus and blood is lower. In addition, since D-Glu is nearly absent in these tissues, D-Gln is likely to be a product of an active D-Glu reaction or removal pathway [56]. D-tyrosine (D-Tyr), D-threonine (D-Thr), D-isoleucine (D-Ile), D-proline (D-Pro), and D-phenylalanine (D-Phe), have been investigated for their activity in the CNS [57,58]. Specifically, D-Ile has been reported to enhance D-Ser release in neurons by regulating the alanine–serine–cysteine 1 (Asc1) transporter [59]. D-Pro has been observed in CNS in mice, specifically in the pituitary and pineal glands [53,60,61]. While D-Pro can be synthetized from L-Pro by proline racemase in prokaryotic and unicellular eukaryotic organisms, there is no literature on its presence in mammals. The previously mentioned D-AAs could be taken up from the gut to reach the systemic circulation and various tissues where they might be metabolized by D-amino acid oxidase. However, some D-AAs are also produced endogenously.

#### 4.2.2. Endogenous D-Amino Acids

Free D-Asp has been detected in the CNS and almost all peripheral tissues of rodents and humans during embryonic development, even exceeding the concentration of the corresponding L-form. It has been found primarily in neurons, in both cytoplasm and fiber tracks, but not in glial cells [16]. In the neonatal cerebral cortex of rats, the transient emergence of D-Asp was demonstrated in the hindbrain, spreading into the forebrain, and then dropping drastically at birth to remain constantly low throughout life. Indeed, in the adult cerebrum it is present in trace amounts only. This decrease with aging suggests the involvement of D-Asp in neuronal development [12,43,62]. In contrast, high concentrations of D-Asp in mature rats and humans have been observed in the neuroendocrine system, including the pineal and pituitary glands, serving as a regulator of hormonal synthesis and secretion [63,64]. D-Asp has been recognized as a cell-to-cell signaling molecule; it is synthesized in neurons, stored in synaptic vesicles of axon terminals, released upon neuronal depolarization in a calcium-dependent manner, interacts specifically on the postsynaptic membrane, and triggers signal transduction. As a result, physiological signaling from one neuron to another is inducted, similarly to other well-known neurotransmitters [62,63,64,65]. However, the nature of D-Asp receptors still needs to be described [64].

The most investigated D-AA is D-Ser, which has mainly been detected in the mammalian forebrain, with the highest concentrations in the cerebral cortex, hippocampus, hypothalamus, and striatum. However, its concentration in different areas of the rodent brain varies with age. The D-Ser level increases by the third postnatal week in the mouse cerebrum, remaining rather constant thereafter, whereas in humans, it stays constant until adulthood but decreases towards half of its original concentration in aged brains. Additionally, in the rodent cerebellum, D-Ser increases until a peak in the second postnatal week; afterwards, it declines drastically to only trace levels [60]. The most important role of D-Ser is related to synaptic neurotransmission. Glutamate is a key neurotransmitter that permits the activation of NMDARs [66]. These receptors have been investigated in relation to neuronal migration, synaptic maturation, synaptic plasticity, learning, memory, and various neural diseases [67]. NMDARs need a co-agonist in addition to glutamate to be activated [68]. Several endogenous small substances found in the CNS extracellular space directly bind to NMDARs and modulate their channel activity. These include the AAs glycine and D-Ser, the polyamine spermine, and the cations Ca^2+^, Mg^2+^, Zn^2+^, as well as H^+^ protons [69]. Before the discovery of D-Ser in the mammalian brain, glycine was believed to be the sole and perfect candidate because of its abundance in this tissue and high affinity to the binding site. However, the discovery of free D-Ser in the mammalian brain changed the knowledge on NMDARs. D-Ser binds to NMDA receptors, with an affinity up to three times higher than that of glycine, thanks to its enantioselective interaction [70]. At the molecular level, glutamate release triggers receptor activation, while D-Ser behaves as an allosteric modulator [69]. In this sense, the localization of D-Ser in tissues follows the distribution of NMDARs in the brain [60]. Interestingly, D-Asp and D-Ala appear to act as endogenous NMDARs agonists [51,57]. This suggests the existence of mechanisms to control their release and subsequent removal from synapses [71].

Another endogenous D-AA, namely D-Cys, has received particular attention for its putative function in synaptic transmission [15]. This molecule is involved in a novel pathway for H_2_S synthesis, which is an important regulator of physiological functions, for instance, regulating inflammation processes, protecting cerebellar neurons from oxidative stress, and improving the dendritic development of Purkinje cells [15,70,72]. Additionally, H_2_S activates NMDARs and enhances long-term potentiation, suggesting a role in neurotransmission [44,73]. The highest concentrations of D-Cys in mice have been detected in the forebrain, particularly in prefrontal cortex, thalamus, striatum, and hippocampus [74]. However, it has also been found in midbrain and hindbrain areas, such as in the medulla and cerebellum, as well as in CSF. In mice, the concentration of D-Cys follows a similar trend to D-Asp and D-Ser. Indeed, its concentration in the brain is higher at the embryonic stage and then steadily declines, remaining at constant levels during young adulthood [75]. Naturally, the involvement of D-Cys in neurodevelopment has also been investigated in mammals. It is recognized as a negative physiological regulator of neural progenitor cells homeostasis in the developing brain by inhibiting their proliferation along with other growth factors and signaling molecules [76].

### 4.3. D-Amino Acids as Potential Biomarkers

A comprehensive classification of D-AAs based on their in vivo activity can be found in Lewis and Seckler’s review [77]. Many D-AAs have been examined for their presence in the brain, with a significant focus on neurological diseases. There is no evidence yet in the literature of competition between D- and L-enantiomers for the mechanisms of action described. For instance, D-serine stereoselectively binds to NMDA receptors at the glycine binding site, while L-serine has not been linked to the same pathway. These mechanisms can be defined as specific D-amino acid-dependent pathways with defined biological functions and no direct involvement of L-amino acids [78].

It is worth noting that four D-AAs, namely D-Asp, D-Ala, D-Ser, and D-Glu, affect neurotransmission. These biomolecules exert their effect by modulating the NMDARs either at the glutamate (D-Asp/D-Glu) or the glycine binding sites (D-Ala/D-Ser), since both sites need to be occupied to open the NMDA receptor ion channel [61,79]. NMDAR signaling has a broad and significant role in regulating neuronal migration and synaptic development and plasticity, and also in further physiological processes such as sensory/motor integration, learning, and memory [62,80]. Perturbations in NMDAR physiological functions are correlated to the aetiology and phenotype of neurodevelopmental and other brain disorders [47,66,81]. Either hyper- or hypo-activation of NMDARs is involved in neurological disorders, such as AD, stroke, and Huntington’s disease, neuropathic pain, and epilepsy, as well as in neuropsychiatric disorders, namely depression, schizophrenia, drug addiction, and anxiety, and neurodevelopmental conditions including autism spectrum disorder (ASD) [14,62,79,82]. Recent advances suggest that D-AAs could serve as biomarkers for these disorders. D-AAs dysregulation in biological fluids or tissues may reflect changes associated with neurological diseases. Their potential as biomarkers arises from their involvement in critical neurological pathways or their changing levels across different neurodevelopmental stages [77]. Furthermore, several studies have focused on the therapeutic effects of D-AAs in human and animal models. Shi et al. [60] reported an interesting overview on the numerous improvements that have been obtained in many diseases, including neurological disorders, through oral administration or injection of these molecules.

NMDARs represent a widely expressed class of receptors involved in many organ functions, not only neuronal cellular communication. They are implicated in several cell types regulating important physiological functions in the gut, kidneys, lungs, skin, immune system, and musculoskeletal apparatus [67,68,69]. For this reason, changes in D-AA levels need to be considered within the bigger picture with reference to the gut–kidney–brain axis, also bearing in mind their main gut microbial origin [44]. Further investigations into kidney or gut functions may be extremely interesting but are beyond the scope of this review, which focuses solely on neurological diseases.

#### 4.3.1. D-Serine

Dysfunctions of NMDARs have been associated with fluctuations in D-Ser concentration in the brain [41]. Indeed, a reduction in D-Ser is likely to lead to NMDAR hypofunction. Conversely, higher concentrations of D-Ser lead to the overstimulation of NMDARs, promoting excitotoxicity [70]. In other words, excessive D-Ser contributes to cells death and damage through excessive influx of calcium. Changes in D-Ser levels have been associated with several brain disorders, such as migraine, epilepsy, amyotrophic lateral sclerosis (ALS), ischemia, depression, PD, Huntington’s disease, schizophrenia, and attention deficit hyperactivity disorder (ADHD) [41,71].

Increased D-ser may contribute to NMDAR hyperactivity, excitotoxicity, and neurodegeneration. For example, D-Ser enhances aberrant NMDAR activation in migraine [83], and inborn errors of its biosynthesis can have a role in epilepsy [14]. Furthermore, in epileptic patients, an increase in D-Ser content in the cortical temporal lobe has been registered, while in a rat model for epilepsy, its concentration was lower in the hippocampus [84,85]. In patients with sporadic/familial ALS, D-Ser levels are increased in the spinal cord, causing motoneuron degeneration by overstimulating NMDARs [86].

Increased brain and CSF D-Ser levels have been demonstrated in clinical and preclinical studies on AD. D-Ser might constitute a novel potential biomarker for early AD diagnosis [72]. AD is characterized by deposition of amyloid plaques in the brain, i.e., the accumulation of amyloid-β (Aβ) oligomers, causing a local increase in D-Ser concentration [74]. Further studies should elucidate the correlation between Aβ neurotoxicity and the amount of D-Ser, since the mechanism behind the impairment of NMDAR due to Aβ peptides is still unclear [75,76]. AD pathology is typically associated with cerebrovascular pathology; interestingly, in cerebral ischemia, human CSF is enriched in D-Ser [87]. Moreover, a correlation between depression and AD neuropathology has been suggested. These two disorders share common outcomes, including lower synapse density and depressive-like symptoms [88]. In depression, an increase of D-Ser in plasma has been detected, but the link between amyloid accumulation and late-life depressive symptoms still needs to be investigated [89].

A significant reduction in serum and CSF levels of D-Ser in patients with schizophrenia has been demonstrated. D-Ser has also been administered in combination with antipsychotics for the treatment of schizophrenia and in particular, treatment-resistant schizophrenia [90,91]. The administration of D-Ser for schizophrenia has been examined also in murine models, showing improvements in learning and memory tasks [92] and also a trend towards inversion of the natural age-related decline in cognitive flexibility, large-scale functional brain connectivity, and neuronal morphology and plasticity, including dendritic spine density [93]. Indeed, in addition to the pathological conditions that lead to alterations in D-Ser concentration, natural age-related cognitive deficits in the hippocampus have been linked to reductions in D-Ser levels because of a decrease of serine racemase expression in the rat hippocampus. This reduction is associated with impaired NMDAR-dependent synaptic plasticity, which is crucial for cognitive function [94,95]. However, this is still controversial, since in other brain areas, no changes are detected [94].

In patients affected by PD, increases in D-Ser in CSF and the post mortem caudate putamen have been observed, suggesting a link between nigrostriatal dopaminergic degeneration and D-Ser upregulation [96]. Interestingly, in patients affected by Huntington’s disease, D-Ser concentration is lower than in healthy controls [87]. Interestingly, in a rat model for ADHD, a decrease in D-Ser levels in the medial prefrontal cortex was obtained, suggesting that the typical inattention and hyperactivity associated with this disorder may be due to an abnormal D-serine metabolism underlying dysregulation of dopamine and NMDARs [97].

#### 4.3.2. D-Aspartate

The involvement of D-Asp in NMDAR modulation and neurotoxicity and its putative role as a drug target for neurological diseases have been described in several rodent models [98,99,100,101,102]. As mentioned above, free D-Asp concentration in mammals peaks in embryos and it drastically decreases at birth [64]. Further studies are still needed to investigate the role of the transient emergence of this metabolite in the embryonic phase. At that stage, neurogenesis and the connectivity of neuronal circuitry are controlled and regulated by NMDARs; thus, a deficiency in D-Asp might have a significant impact on fetal neuronal arrangement. As a result, a correlation between incomplete physiological brain development and the occurrence of psychotic-like symptoms in adulthood might be considered [103]. Increased D-Asp residues in proteins in AD brains have been reported [103]. In contrast, severe reduction in free D-Asp concentration and downregulation of NMDARs were detected post mortem in the prefrontal cortex and striatum of patients with schizophrenia [104,105]. Lower concentrations of D-Asp have also been observed in the substantia nigra of PD patients [106]. Finally, a mouse model for ASD exerted altered D-Asp metabolism, including increased D-Asp content in the prefrontal cortex, hippocampus, and serum, suggesting the need for human cohort studies to further investigate its role in ASD [14].

#### 4.3.3. Other D-Amino Acids

D-Ala has been investigated in both schizophrenia and AD, because of its role in modulating NMDARs [15,107,108]. In AD patients, higher concentrations of D-Ala were registered in the gray matter of post mortem brains [108,109]. In contrast, in a rodent model for AD, decreased amounts in AD plasma were reported compared with the healthy controls [109].

Other D-AAs showing similar trends are D-Leu and D-Pro; their concentration in plasma in a rat model for AD suggests their involvement in the disease and their putative role as plasma-based biomarkers. Conversely, D-Phe levels were found to be drastically increased in AD rat plasma [109]. Additionally, increases in D-Pro in human plasma have been determined in mild cognitive impairment (MCI) and dementia [110].

D-Glu has also been related to AD, because of its decreased presence in the hippocampus, hence leading to reduced activation of NMDARs and worsening of symptoms [77].

A schematic overview of previously described changes in D-AA concentrations related to brain diseases in mammals is given in Table 1.

**Table 1:** Alterations of D-amino acid levels recorded in biological samples from animal models or patients affected by neurological disorders. Symbols: ↓, decrease in concentration; ↑, increase in concentration.

D-Amino Acid	Matrix	Alterations	Species	Neurological Diseases	Refs.
D-serine	blood, plasma, serum	↓	humans	schizophrenia	[111,112,113]
	serum	↑	humans	bipolar disorder, Alzheimer’s disease, major depressive disorder	[114,115,116]
	cerebrospinal fluid, serum	↑	humans	Alzheimer’s disease	[75,115,117]
	hippocampus	↓	rats	substances use disorder	[118]
	spinal cord	↑	humans, mice	amyotrophic lateral sclerosis	[12,86]
	cerebrospinal fluid	↓	humans	schizophrenia, major depressive disorder, Parkinson’s disease	[106,119]
	substantia nigra	↓	macaques	Parkinson’s disease	[106]
	striatum	↑	monkeys	Parkinson’s disease	[120,121]
	cerebrospinal fluid	↑	humans	Parkinson’s disease	[121]
	post mortem caudate putamen,	↑	humans	Parkinson’s disease	[96]
	plasma	↓	humans	Huntington’s disease	[87]
	cortical temporal lobe	↑	humans	intractable epilepsy	[84]
	hippocampus	↓	rats	temporal lobe epilepsy	[85]
	cerebrospinal fluid	↑	piglets, humans	global cerebral ischemia	[122]
	medial prefrontal cortex	↓	rats	ADHD	[97]
D-aspartate	prefrontal cortex, hippocampus, serum	↑	mice	autism spectrum disorder	[99]
	gray matter	↑	humans	Alzheimer’s disease	[56]
	plasma	↓	rats	Alzheimer’s disease	[109]
	prefrontal cortex, striatum	↓	humans	schizophrenia	[98,105]
D-glutamate	hippocampus, serum	↓	humans	Alzheimer’s disease	[77,107]
D-alanine	gray matter	↑	humans	Alzheimer’s disease	[46,108]
D-proline	plasma	↑	humans	mild cognitive impairment, dementia	[110]
	plasma	↓	rats	Alzheimer’s disease	[109]
D-alanine	plasma	↓	rats	Alzheimer’s disease	[109]
D-leucine					

## 5. Chiral-Targeted Metabolomics

Metabolomics provides the opportunity to better understand the mechanisms underlying various diseases. Since the metabolome is the signature of all the complex processes that take place in living beings, fluctuations in metabolite profiles in biological samples in health and disease can be studied as indicators of pathological dysfunctions prior to the onset of symptoms or even disease development. Targeted metabolomics methods are typically developed for the accurate absolute quantification of predefined metabolites. Limited numbers of metabolites are selected in advance and tandem MS analysis in multiple reaction monitoring (MRM) mode is carried out. Brain metabolomics studies are frequently performed on post mortem tissues, but also on limited-volume biological samples from living animals. Indeed, much interest has been shown among scientists regarding in vivo brain metabolomics techniques in small animal models, which result in sample volumes from the picoliter level to about 50 µL [35]. The major types of matrices investigated in this field comprise biofluids, including CSF, plasma, serum, ECF, and post mortem brain tissues [5,34]. Moreover, there are wide differences between D- and L-AAs concentrations in these matrices. D-enantiomers are physiologically present in mammals in very low amounts, frequently even in trace levels [123]. This makes their detection and quantification even more challenging, requiring methods with very high sensitivity and selectivity. In this section, the major advances in targeted chiral brain metabolomics for the enantioselective analysis of D-AAs through LC-MS(/MS) are discussed.

### 5.1. Stereospecific Recognition

Living organisms possess enzymes that stereospecifically recognize, metabolize, and interconvert enantiomeric isomers of molecules. Likewise, accurate enantioselective analysis is a pivotal step for distinguishing and individually quantifying D- and L- forms of AAs in real-world samples, which frequently consist of complex and heterogeneous matrices. The separation of enantiomers poses a challenge owing to the necessity of prior enantioselectivity. Furthermore, multiplexed detection, i.e., the simultaneous characterization and quantification of multiple metabolites, is becoming increasingly important in clinical settings to enhance disease specificity [15].

Different techniques for enantioselective analysis based on stereospecific recognition can be exploited. Current tools rely on capillary electrophoresis (CE) [124] and chromatography, including (ultra) high-performance liquid chromatography ((U)HPLC) [125], gas chromatography (GC) [126], and supercritical fluid chromatography (SFC) [127]. Moreover, spectroscopic and spectrometric technologies are also frequently applied in this field such as NMR [128] and more recent ion-mobility spectrometry (IMS) techniques [129,130]. This review focuses only on liquid chromatography coupled to mass spectrometric detection.

Chiral chromatographic separation strategies typically fall into two categories: direct or indirect methods [131]. The first type includes the formation of transient diastereomeric complexes between the solute and a chiral selector [132]. These chiral selectors can either be dissolved into the mobile phase or immobilized on the stationary phase [133]. In the first case, crown ethers or cyclodextrins are employed and the separation takes place on an achiral column through the formation of diastereomeric adducts with the enantiomeric analytes in solution [134]. Instead, chiral selectors, which are bound to the stationary phases, are polysaccharides, like cellulose and amylose derivatives, macrocyclic antibiotics, or cyclodextrins, and provide direct separation on the column [135,136].

**Figure 1:**
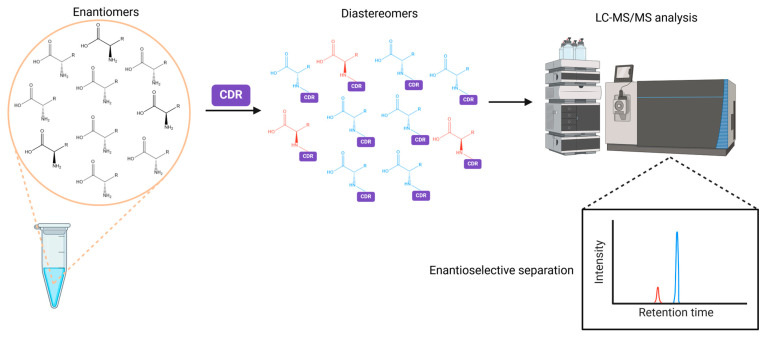
General analytical workflow for the enantioselective LC-MS/MS analysis of amino acids in biological samples upon upon chemical derivatization with a chiral reagent. Created in BioRender. Lella, C. (2024) https://BioRender.com/d91f759 (accessed on 26 September 2024). Abbreviations: CDR, chiral derivatization reagent; LC, liquid chromatography; MS, mass spectrometry.

Conversely, indirect methods rely on the conversion of enantiomeric pairs into diastereomeric pairs through a derivatization reaction with a chiral reagent, which precedes chromatographic separation on an achiral column. Unlike enantiomers, diastereomers present different physicochemical properties and they can thus be readily differentiated and separated without a chiral environment. The CDR should be composed of (i) a reactive functional group specific to the target analyte, (ii) a chiral moiety for diastereomer formation, and (iii) a detection tag, which can be a chromophore group for UV or fluorescence detection or a signature fragment for MS detection to increase sensitivity [10]. A general analytical workflow for the enantioselective LC-MS/MS analysis of amino acids in biological samples upon chiral derivatization is illustrated in Figure 1.

### 5.2. Chemical Derivatization for the Enantioselective Analysis of D-Amino Acids

Several improvements in targeted chiral brain metabolomics have been achieved thanks to the pre-column chemical derivatization of samples, which enhances the capabilities of LC-MS-based analytical platforms in terms of metabolite stability as well as resolution, efficiency, and selectivity of analysis [137]. The choice of the CDR relies on the chemistry of the metabolites under investigation, as each CDR targets a specific chemical functional group for the formation of the derivatized metabolites through a new covalent bond [138].

Derivatization strategies introduce a new moiety or tag onto the original molecule, providing several beneficial effects. For instance, AAs are characterized by high polarity and hydrophilicity, resulting in poor retention on a reversed-phase LC column [139]. By introducing a hydrophobic substituent into the amino group, retention of the labelled metabolites can be increased on common RPLC columns. Accordingly, improvements in detectability of low-molecular-weight analytes can be achieved. The mass of the target molecule is enlarged through derivatization, thus overcoming interferences caused by impurities within the matrix or solvent and background noise in general, which are usually in the lower *m*/*z* range. This can translate into better detectability of low-molecular-weight metabolites [10]. Furthermore, with electrospray ionization (ESI) sources, ionization efficiency can also be drastically enhanced through the introduction of permanently charged or easily ionizable groups as well as ameliorated analyte nebulization at the LC-MS interface. In this sense, since the retention of derivatized metabolites in RPLC is increased, an organic solvent-rich mobile phase improves analytes’ desolvation from the droplets. Meanwhile, the hydrophobic moiety leads molecules more effectively towards the surface of the droplets, decreasing surface tension and making ion desolvation faster and more efficient [138]. Derivatization can also be beneficial from the point of view of molecular stability. Among AAs, for instance, cysteine quickly and spontaneously undergoes oxidation in biological systems, but this can be effectively avoided through pre-column derivatization of this unstable group [10,140]. In addition, some AAs, including serine and threonine, also present alcoholic groups that behave as nucleophiles in basic conditions. Some reagents can simultaneously derivatize amine and alcoholic groups, allowing the derivatization of amino acids and hydroxy acids in one reaction, for instance [138]. From a mass spectra interpretation perspective, the occurrence of a tag on molecules also improves their fragmentation patterns, allowing quantification in MRM mode, even in trace analysis [137,138].

However, some properties must be taken into account in the choice of CDR. The optical (and chemical) purity of the reagent must be verified, and it should also be available in both enantiomeric forms. Since in mammals D-AAs are present in low concentrations, it is beneficial for accurate quantification to elute them first before the respective L-AAs. This can be obtained by switching CDR configuration and thus reversing the elution order of the DL-AAs. In this way, the influence of tailing phenomena can be avoided, allowing the accurate integration of peaks. In addition, the derivatization reaction should preferentially occur in mild conditions, avoiding side reactions or racemization processes, to preserve the stereochemical integrity and chemical stability of derivatized analytes throughout the entire analytical workflow [10].

The indirect approach of chiral resolution provides a further advantage for quantification purposes. In LC-MS-based metabolomics, matrix effects can influence the analysis outcomes. Isotope-coded derivatization (ICD) can offer a way to solve these issues and ensure accuracy and precision of the method, overcoming the need for an internal standard (IS) for each analyte under study, which would be practically and financially tricky especially when the analysis involves numerous compounds. With ICD, analyte standards are labelled with a stable isotope-coded moiety and can be used as ISs [141]. Either differential or absolute analysis can be performed through ICD. Differential analysis is carried out by derivatizing one sample (e.g., from a healthy control) with the light form of the reagent (i.e., labelled with ^1^H, ^12^C, ^14^N, or ^16^O), whereas another sample (e.g., from a diseased subject) is derivatized with the heavy reagent (i.e., containing ^2^H, ^13^C, ^15^N, or ^18^O). Equal volumes of these derivatized solutions are mixed and then subjected to LC-MS(/MS) analysis. Since light- and heavy-labelled metabolites exert nearly identical properties, they co-elute from the chromatographic column. The mass spectrum, however, displays a pair of peaks with a mass shift corresponding to the difference in mass of the two isotopic labels. The ratio of peak intensities for each pair yields the relative concentration of each metabolite between the two solutions. Comparing the peak areas of the two derivatives, the relative quantification of the metabolite enantiomer is obtained. Conversely, for absolute quantification, the heavy reagent is used to derivatize known concentrations of analytes standards, which take on the role of ISs [142,143].

While employing isotope-coded reagents offers significant advantages for chiral resolution and quantification, some associated issues must be carefully considered. Multi-deuterated reagents may suffer from a chromatographic isotopic effect, where the differences in hydrophobic interactions between hydrogen and deuterium atoms with the stationary phase lead to discrepancies in elution profiles. To mitigate this effect, deuterium atoms can be located as close as possible to the hydrophilic groups. Alternatively, isotopes like ^13^C, ^18^O, or ^15^N, which do not exhibit such effects, can be utilized. Furthermore, reactivity, abundance, and chemical and enantiomeric purity must be evaluated for both light and heavy forms of the isotopic-coded reagent. Thus, expected reactions with superimposing kinetics should be achieved for both forms of the ICD reagent to ensure consistency and reliability in results [141].

Bogos et al. [13] reported a comprehensive list of CDRs used in the derivatization of chiral AAs for enantioselective analysis. Samples has to be subjected to the derivatization reaction with only one enantiomeric form of CDR at a time. Performing the reaction with the other enantiomer, an inversion in the elution order of D- and L-amino acids is expected. Additionally, when the separation of the formed diastereomers is accomplished, comparing the elution sequences obtained using both (+) and (−) enantiomers of the CDR, it is possible to discriminate chiral and achiral derivatives, since these latter provide only one peak in each chromatogram. This can be a useful expedient for confirming the quality and success of the achieved enantioselective chromatographic separation [10].

### 5.3. LC-MS Methods for the Enantioselective Analysis of D-Amino Acids upon Chemical Derivatization

Enantioselective analysis, through either direct or indirect methods, can be carried out on LC-MS platforms, in which diastereomers are separated according to their different physicochemical properties [10]. MS-based methods are extremely useful in brain metabolomics studies.

Numerous methods for the enantioselective separation of chiral biomolecules through direct approaches rely on multi-dimensional LC-MS/MS analysis. Samples are often first derivatized with an achiral reagent, which provides molecules with more lipophilic moieties and higher molecular weights to reduce interferences and increase derivatives’ stability, since these metabolites are often polar and hydrophilic molecules, and also with a favorable detection tag [10,140]. For this purpose, several papers have reported the use of 4-fluoro-7-nitro-2,1,3-benzoxadiazole (NBD-F) as an achiral reagent in metabolomics, even for non-enantioselective analysis [144]. Afterwards, the actual separation is performed through chiral stationary phases via multi-dimensional HPLC or LC-MS/MS techniques [145]. Indeed, NBD derivatives are separated as racemic mixtures in the first dimension on an achiral reversed-phase column, typically of the C18 type. Then, the actual enantioselective separation is obtained on a chiral stationary phase, such as on Pirkle-type columns, followed by MS/MS or fluorometric detection, since NDB-F is suitable as a chromophore [146,147]. Another achiral derivatization reagent is 6-aminoquinolyl-N-hydroxysuccinimidyl carbamate (AQC), which has been used in combination with LC-MS/MS analysis on either quinine- or quinidine-type columns, characterized by chiral zwitterionic stationary phases based on *Cinchona* alkaloids [148,149,150].

Derivatization strategies for the indirect method, as already explained, rely on a pre-column reaction with a chiral compound, followed by LC-MS(/MS) analysis. The chromatographic separation can be achieved on a common achiral RPLC column, on which the derivatized analytes show retention. In contrast, the choice of achiral derivatization for enantioselective purposes implies the consequent use of a chiral column or a combination of an achiral and chiral column, such as in multiple heart cutting (MHC) 2D-LC separations. In these set-ups, selected peaks of interest that elute from achiral 1D separation (usually RPLC) are transferred to chiral 2D separation without interfering components and matrix effects, through an interface equipped with multiple sampling loops for fraction storage [10,151,152]. In addition, a single LC-MS run is often not sufficient to obtain the simultaneous detection of several metabolites, due to the similar physical and chemical properties of enantiomers and the trace levels of D-AAs, for instance [10]. Pre-column chiral derivatization, in this sense, can offer a valid alternative in terms of time, efficiency, and costs of the analysis.

#### Chemical Derivatization for Indirect Enantioselective Analysis

In this paragraph, the optimized methods using CDRs for the enantioselective analysis of amino acids in biological matrices are discussed (Table 2).

Yamamoto et al. [153] used commercially available Nα-(5-fluoro-2, 4-dinitrophenyl)-L-leucinylamide (L-FDLA) in combination with NaHCO_3_ as an alkaline environment, entailing a desalting step before analysis to avoid ion suppression effects. Simultaneous analysis of 10 DL-AAs was obtained with this method, which was applied to mouse cortex, cerebellum, hippocampus, and thalamus samples for studying fluctuations in metabolite levels in aged brains. The instrumental analysis was carried out on an HPLC system with a C18 column coupled to triple quadrupole MS in MRM mode through ESI+ [153]. L-FDLA with the addition of volatile triethylamine (TEA), instead of NaHCO_3_, to avoid the desalting step was also employed by Kobayashi et al. [154] for the development of an enantioselective method to separate 17 chiral AAs. Again in this case, chromatographic separation was achieved on a HPLC system (C18 column) coupled with a triple quadrupole (TQ) MS using positive atmospheric pressure ionization. The optimized method was then applied to human HepG2 cells and culture medium, and D-Asp and D-Ser were selectively detected and quantified [155]. Moreover, Liu et al. [156] obtained the simultaneous separation and identification of 14 DL-AAs in human serum on a conventional C18 column after derivatization with commercially available L-FDLA in combination with TEA. Identification of all analytes was achieved by tandem MS detection with ESI+ in MRM mode.

L-FDLA is a modified version of the so-called “Marfey’s reagent”, 1-fluoro-2,4-dinitrophenyl-5-L-alanineamide (L-FDAA); the substitution of L-alanine with L-leucine improves the sensitivity and resolution of DL-AAs [157]. Ogunkunle et al. [158] compared 1-(9-fluorenyl)-ethyl chloroformate (FLEC reagent) and L-FDAA as CDRs for the quantification of chiral AAs secreted by murine islets of Langerhans in response to low- and high-glucose stimulation. The effectiveness of both reagents in separating chiral AAs was first evaluated in islet-specific buffers. Because of the inability to achieve baseline resolution of FLEC-derivatized AAs, biological samples were then analyzed using the optimized method with Marfey’s reagent. The simultaneous separation and quantification of 13 DL-AAs was performed on a UHPLC system with a C18 column coupled through ESI+ to a TQ-MS in MRM mode. The release of chiral AAs in rodent islets of Langerhans was also investigated by Lee et al. [159], with another modified Marfey’s reagent. After precolumn derivatization using Nα-(2,4-dinitro-5-fluorophenyl)-L-valine amide (L-FDVA), LC−TQ-MS/MS-MRM analysis was performed with a phenyl-hexyl stationary phase in ESI negative mode.

Visser et al. [160] investigated several CDRs and found that (S)-N-(4-nitrophenoxycarbonyl) phenylalanine methoxyethyl ester ((S)-NIFE) gave the best performances in terms of sensitivity but also required only mild derivatization conditions and had good stereoselective properties. They consequently achieved in a single run the separation and quantification of 19 DL-AAs in human CSF, urine, and plasma. Their method was performed on a UHPLC system equipped with a C18 column and coupled to a TQ-MS. The analysis was carried out in ESI+ mode. Similar workflows have also been applied for the quantification of the D-AAs in rodents. Xing et al. [109] obtained the simultaneous determination of 18 D-AAs in rat plasma, and Li et al. [161] extended their study to rat tissues, including the cerebral cortex, hippocampus, cerebellum, and olfactory bulb, for the absolute quantification of 11 DL-AAs.

Another CDR that has been studied is o-phthalaldehyde (OPA), which forms fluorescent isoindole derivatives in combination with chiral nucleophilic thiols. The most used of these are N-acetyl-L-cysteine (NAC), N-acetyl-D-penicillamine (NAP), N-(tert-butoxycarbonyl)-L-cysteine methyl ester (NBC), N-(tert-butylthiocarbamoyl)-L-cysteine ethyl ester (BTCC), isobuteryl-L-cysteine (IBLC), and N,N-dimethyl-L-cysteine (DiCys) [162]. Müller et al. [163] developed an efficient UHPLC method involving fluorescence detection for the enantioselective analysis of derivatized AA standards. However, due to the complexity of biological matrices and sensitivity issues, they preferred to turn towards mass spectrometry detection. They reported precise discrimination of serine, alanine, and methionine enantiomers in mouse gut and human serum, plasma, and urine with their UHPLC-ESI-QToF-MS/MS set-up, based on diastereomer separation on a common C18 column.

The OPA reagent has recently been used in targeted metabolomic studies in combination with NAC for investigating fluctuations in serine enantiomers homeostasis related to PD. After precolumn derivatization, through RPLC analysis, D- and L-Ser levels were studied in human serum, CSF, post mortem caudate putamen, and superior frontal gyrus, and the medial prefrontal cortex, midbrain, and striatum from monkeys and mice [96,121,164,165].

Novel chiral derivatization reagents based on triazine moieties, such as 1-(4,6-dimethoxy-1,3,5-triazine-2-yl)pyrrolidine-2carboxylate (DMT-(S)-Pro) and 2,5-dioxopyrrolidin-1-yl-1-(4,6-dimethoxy-1,3,5-triazine-2-yl) pyrrolidine-2-carboxylate (DMT-(S)-Pro-OSu), have been synthesized. The latter allowed separation of 19 DL-AAs in a single run, after trying several elution gradient profiles and different columns. The method was applied to human saliva, detecting most of the D-AAs at trace levels [166].

For AAs containing a thiol functional group, a phosphorus-based novel reagent has been developed, namely (R)-(5-(3-isothiocyanatopyrrolidin-1-yl)5-oxopentyl) triphenylphosphonium (NCS-OTPP). This compound allowed the separation of DL-cysteine and DL-homocysteine in human saliva using a C18 UHPLC-Orbitrap MS/MS method [167]. Organic phosphorus chemistry was also involved in the so-called SIPAL strategy. The N-hydroxysuccinimide ester of N-diisopropyl phosphoryl L-alanine (DIPP-L-Ala-NHS) with a stable isotopic label on the oxygen, used as CDR, enabled Zhang et al. [168] to separate chiral amino-containing metabolites in human urine.

Huang et al. [169] developed a novel C18 HPLC-TQ-MS/MS method preceded by derivatization with 1-benzoyl-pyrrolidine-2-carboxylic acid 5-cloro-2-formylphenyl ester (D-BPCl) to simultaneously separate and quantify 29 AAs in 24 min, including 12 proteinogenic and two nonproteinogenic AA enantiomeric pairs and the achiral glycine. Their method was then applied for the targeted and pseudo-targeted analysis of chiral amino-containing metabolites in human urine in the context of gastric cancer.

A CDR that has gained more attention in the past few years is diacetyl-D-tartaric anhydride (DATAN). Interestingly, DATAN undergoes reactions on either amine or hydroxy functional groups, enabling the simultaneous analysis of chiral AAs and hydroxy acids (HAs) in a single analytical run. Pandey et al. [170] developed a novel strategy for untargeted chiral metabolomics of metabolites containing these groups by chiral derivatization coupled with C18 LC-ESI(+)-Orbitrap-MS/MS analysis in all-ion fragmentation (AIF) acquisition mode. Unlike MRM, AIF provides the systematic fragmentation of all ionized analytes without requiring any prior precursor scan or selection. This strategy allows a broader examination of the sample, highlighting its value in untargeted metabolomic studies [171]. The method was validated with the separation of 19 DL-AAs and 9 DL-Has; afterwards, the D-enantiomeric forms of 22 AAs and 8 HAs in bone marrow and 20 AAs and 6 HAs in blood plasma were detected. It is worth noting that this method allowed the detection of achiral metabolites as well.

Derivatized AAs are generally separated by RPLC. Nevertheless, hydrophilic interaction liquid chromatography (HILIC) can be a suitable alternative because of the high hydrophilicity of the analytes of interest. Sakamoto et al. [172] obtained efficient separation of alanine and serine enantiomers in rat plasma after derivatization with succinimidyl-(4S)-(3-[(benzyloxy)carbonyl]-5-oxo-1,3-oxazolidin-4-yl)acetate ((S)-COXA-OSu) on a HILIC-ESI(-)-TQ-MS/MS set-up. In this set-up, D-amino-acid derivatives eluted faster than the corresponding L-derivatives using (S)-COXA-Osu.

The multiplexed enantioselective analysis of all proteinogenic D-AAs in biological samples is still challenging. At present, none of these CDRs enable an ultimate complete strategy. For instance, DATAN achieved the enantioselective separation of the highest number of DL-AAs and DL-HAs in a single analytical run with an untargeted approach. It might be interesting to focus on switching to a targeted study for the quantification of not only D-AAs but also D-HAs, since they have been involved in metabolomic studies for biomarker discovery. D-HAs have been investigated in metabolic diseases such as diabetes. Indeed, alterations in DL-lactic acid and DL-3-hydroxybutyric acid concentrations have been observed in diabetes mellitus and diabetic acidosis. Also, D-2-hydroxyglutarate and L-2-hydroxyglutarate are recognized as “oncometabolites” because of their involvement in the formation and malignant progression of gliomas and acute myeloid leukemia [173,174]. Tsutsui et al. [175] carried out the simultaneous determination of trace amounts of DL-lactic acid and DL-3-hydroxybutyric acid enantiomers in the saliva of diabetes mellitus patients through UHPLC-ESI(+)-TQ-MS/MS upon chemical derivatization with (S)-1-(2-pyrrolidinylmethyl)-pyrrolidine (S-PMP). Thereafter, they tested another CDR, namely (S)-1-(4,6-dimethoxy-1,3,5-triazin-2-yl)pyrrolidin-3-amine (DMT-3(S)-Apy), achieving the separation of 13 chiral and achiral carboxylic acids in saliva from healthy volunteers and type-II diabetes patients [176].

It is worth noting that most of the methods employed for the investigation of D-AAs and D-HAs rely on ESI+, even though easier ionization of the carboxylic acid functionality on these molecules would be expected with ESI-. In this sense, the sensitivity of carboxylic acids in ESI- is insufficient for their analysis in complex matrices, including plasma and urine [170,176]. Further studies on this aspect might be interesting, even on other matrices such as CSF and ECF.

**Table 2:** LC-MS(/MS) methods developed for the indirect enantioselective analysis of chiral amino acids in biological samples.

Matrix	Species	CDR	Analytical Technique	Metabolites	Ref.
cortex, cerebellum, hippocampus, and thalamus	mice	L-FDLA	RPLC-ESI(+)-TQ-MS/MS	10 DL-AAs	[153]
hepatoma-derived cell line, HepG2	humans	L-FDLA	RPLC-ESI(+)-TQ-MS/MS	D-Ser and D-Asp	[155]
serum	humans	L-FDLA	RPLC-ESI(+)-TQ-MS/MS	14 DL-AAs	[156]
islets of Langerhans	mice	L-FDAA	RPLC-ESI(+)-TQ-MS/MS	13 DL-AAs	[158]
islets of Langerhans	mice	L-FDVA	RPLC-ESI(−)-TQ-MS/MS	D-Ser, D-Ala, D-Asp	[159]
plasma	rats	(S)-NIFE	RPLC-ESI(+)-TQ-MS/MS	18 DL-AAs	[109]
CSF, plasma, urine	humans	(S)-NIFE	RPLC-ESI(+)-TQ-MS/MS	19 DL-AAs	[160]
hippocampus, cerebral cortex, olfactory bulb, cerebellum	rats	(S)-NIFE	RPLC-ESI(+)-TQ-MS/MS	11 DL-AAs	[161]
serum, plasma, urine	humans	OPA/IBLC	RPLC-ESI-QToF-MS/MS	DL- serine, alanine, methionine	[163]
saliva	humans	DMT-(S)-Pro-OSu	RPLC-ESI(+)-TQ-MS/MS	19 DL-AAs	[166]
saliva	humans	NCS-OTPP	RPLC-ESI(+)-Orbitrap-MS/MS	DL-Cys, DL-HCy	[167]
urine	humans	DIPP-L-Ala-NHS	RPLC-ESI(+)-Orbitrap-MS/MS	Chiral amino-containing metabolites	[168]
urine	humans	D-BPCl	RPLC-ESI-TQ-MS/MS	Chiral amino-containing metabolites	[169]
bone marrow (BM) and peripheral blood (PB) plasma	humans	DATAN	RPLC-ESI(+)-Orbitrap-MS/MS	22 DL-AAs, 8 DL-HAs in BM; 20 DL-AAs, 6 DL-HAs in PB	[170]
plasma	rats	(S)-COXA-OSu	HILIC-ESI(−)-TQ-MS/MS	DL-alanine and serine	[172]

Abbreviations: DL-AAs, DL-amino acids; D-BPCl, 1-benzoyl-pyrrolidine-2-carboxylic acid 5-cloro-2-formylphenyl ester; DATAN, diacetyl-D-tartaric anhydride; DIPP-L-Ala-NHS, N-hydroxysuccinimide ester of N-diisopropyl phosphoryl L-alanine; DMT-(S)-Pro-OSu, 2,5-dioxopyrrolidin-1-yl-1-(4,6-dimethoxy-1,3,5-triazine-2-yl) pyrroli-dine-2-carboxylate; ESI, electrospray; HILIC, hydrophilic interaction liquid chromatography; L-FDAA, 1-fluoro-2,4-dinitrophenyl-5-L-alanineamide; L-FDLA, Nα-(5-fluoro-2, 4-dinitrophenyl)-L-leucinylamide; L-FDVA, Nα-(2,4-dinitro-5-fluorophenyl)-L-valine amide; MS, mass spectrometry; NCS-OTPP, (R)-(5-(3-isothiocyanatopyrrolidin-1-yl)5-oxopentyl) triphenylphosphonium; OPA/IBLC, o-phthalaldehyde/isobuteryl-L-cysteine; RPLC, reversed-phase liquid chromatography; (S)-COXA-OSu, succinimidyl-(4S)-(3-[(benzyloxy)carbonyl]-5-oxo-1,3-oxazolidin-4-yl)acetate; (S)-NIFE, (S)-N-(4-nitrophenoxycarbonyl) phenylalanine methoxyethyl ester; TQ, triple quadrupole.

## 6. Conclusions

Targeted chiral brain metabolomics is becoming more and more important in biomedical studies. Much interest has been shown in the interaction of D-AAs with NMDARs. Hypo- and hyper-activation of these receptors are implicated in neuropathological pathways of several diseases, such as AD, schizophrenia, and epilepsy. Given the role of these metabolites in modulating NMDARs and influencing neurotransmission, new insights about neurological diseases and their causes can be obtained by studying fluctuations in D-AAs levels under pathophysiological conditions. However, D-AAs are naturally present at trace levels in mammals; thus, their detection and quantification require highly sensitive and selective analytical platforms. Additionally, these metabolites are characterized by the presence of chirality; hence, enantioselective analysis is mandatory.

In this review, the most used strategies for assessing targeted chiral brain metabolomics using LC-MS/MS have been explored. Stereospecific recognition is achievable by employing chiral selectors or through chemical derivatization using a chiral reagent. The use of chemical derivatization strategies is especially interesting because of the adaptability and versatility that they provide to the analytical method. In LC-MS(/MS) methods, an achiral chromatographic column can be employed after derivatization of the analytes with a chiral reagent, simplifying the analytical process and reducing costs. From an analytical point of view, better sensitivity and selectivity can be achieved. This is crucial in the targeted analysis of biological samples, which are characterized by a high complexity of the matrix as well as by very low concentrations of D-AAs. Several CDRs are currently employed in targeted metabolomics. D- and L-AAs are transformed from enantiomers into diastereomers after reaction with a CDR and therefore, they can be separated on an achiral stationary phase. Because of the high polarity and hydrophilicity of these metabolites, HILIC columns can also be employed for their separation. However, RPLC has been the most used separation mode till now. Through chiral derivatization, hydrophobic moieties are added to the target molecules, enhancing their stability and retention on common RP columns. Derivatization is also beneficial for obtaining more efficient ionization and adding a characteristic fragment that can help in MS spectra interpretation.

Nevertheless, achieving the simultaneous separation of all DL-AAs in a single run remains challenging due to the complex nature of biological samples and typically limited sample volumes. Further studies are needed to develop more efficient LC-MS(/MS) methods and also to improve sampling techniques. For this purpose, for preclinical studies, in vivo microdialysis, cOFM, and SPME may be suitable tools in order to obtain biological samples such as extracellular fluid and cerebrospinal fluid that are more representative of the actual metabolomic profile in the brain, compared with peripheral biofluids. However, for human studies, less invasive sampling techniques are typically applied. Consequentially, metabolomics analysis in clinical studies mainly relies on plasma, blood, urine, and post mortem tissues. Additionally, exploring and developing novel CDRs, alongside optimizing derivatization and analytical conditions, can lead to the detection and quantification of a larger number of DL-AAs in a shorter analysis time and with better sensitivity and specificity. The multiplexed detection of several metabolites can lead to the discovery of novel drug targets and diagnostic tools to make the identification and treatment of neurological diseases more effective and faster, and also to new insights about the biochemical mechanisms underlying certain pathophysiological conditions.

## Data Availability

No new data were created or analyzed in this study. Data sharing is not applicable to this article.
